# Copper Amalgam

**Published:** 1887-12

**Authors:** Geo. H. Weagant

**Affiliations:** Cornwall, Ont.


### COPPER AMALGAM.

READ BEFORE THE CONNECTICUT VALLEY DENTAL SOCIETY AT MONTREAL, P. Q. JULY 22D, 1887.

BY GEO. H. WEAGANT, L. D. S., CORNWALL, ONT.

Copper amalgam is composed of pure copper and pure mercury in variable proportions. The less mercury it contains the more quickly it sets and the harder it becomes. When properly made it is exceedingly pleasant to work, fine-grained and plastic, and sets either slowly or rapidly, as we desire it, and are pleased to prepare it. It becomes very hard—harder in fact than any amalgam made from alloys. It is not known to shrink or expand in the least degree. It does not ball up nor change its shape in any way during the setting or afterwards, and finally, instead of having any injurious effect upon the teeth or surrounding tissues, it is decidedly beneficial to them, acting as an antiseptic or germ destroyer. But, although it does not cause discoloration of the teeth, the filling itself will quickly and emphatically become black—very black—upon the surface. It should always be carefully polished when hard, for, although polishing does not prevent its turning black, it is a polished black, and not so disagreeable and dirty looking as when left with a rough surface. It is, perhaps, also slightly deficient in edge-strength, although of this I am not very positive. It is extremely hard to drill and cut, and whenever there is a probability that it may be necessary, at some future time, to remove the filling, it would be advisable to use some softer amalgam. Immense quantities are used in Europe for filling decayed teeth, but it is comparatively unknown in this country. In fact, very few of our dentists have ever heard of it, and fewer still have seen it. In Europe it is employed chiefly because it is cheap; in America, because it is supposed to possess some qualities which make it, for special cases at least, the best filling known. I have never seen fillings of copper amalgam except those inserted by myself, or by my friends who had used material made and furnished by me. I have not been able to secure a sample of the European article, but Dr. Bogue, of New York, assures me that what is made in England and Germany is very dirty to work, soiling the hands and not giving satisfactory results. I have no doubt it contains some other metal than copper and mercury—probably zinc or oxide of iron.

The literature of the subject is very meagre, just sufficient mention being made of it occasionally, and at long intervals, to intimate that such a thing is actually used by somebody in the dental profession. In the preparation of this paper I have, therefore, had to rely almost entirely upon my own experience in its use.

The first mention I can find of copper amalgam is in the October number of the *Dental Cosmos* for 1859—nearly thirty years ago—but the editor, in a foot-note, says he would not recommend it for filling teeth, as "both of its constituents, copper and mercury, are highly objectionable." In 1874, Dr. Bogue, in a paper on Amalgams, read before the New York Odontological Society, mentions copper amalgam, and says: "It is quite difficult to prepare, is dark in color and its oxide is poisonous. Though extensively used in Germany, it is fortunately seldom or never employed in this country." At that time he had evidently not studied the material in the mouth, though he was then engaged in conducting a series of experiments with a large number of different amalgams, copper among the rest, to determine their physical properties and physiological action upon the human system. In 1875 he issued a pamphlet giving the result of these experiments. He found that copper amalgam, composed of copper precipitated upon mercury, was the only one which did not shrink or expand in the least. But as to its physiological action upon the system, he did not appear to be prepared to speak definitely, so far as copper was concerned. He conclusively proved that the mercury in all amalgams was without deleterious effect upon the system. However, in 1880, he states, in discussing the amalgam question, that he considers copper amalgam to be a preventive of decay where other materials have failed, and looks upon it as next to palladium.* Now, we all know what Dr. Bogue thinks of palladium, and when he declares a thing is next to palladium he could not say more in its favor. He also gives a method for its preparation and manipulation, in which I fancy he must have made an error, as I was unable to prepare it in the same way. Dr. Rogers, of London, England, uses it to a great extent.† Dr. Pritchard, also of London, speaks very highly of Sullivan's cement, which is an amalgam of copper and mercury,‡ and Dr. Bödecker, years ago, saw great numbers of copper amalgam fillings in England; they looked quite black, but preserved the teeth well.§ Dentists there used it for cheap work only, on account of its looking so black, but it saved the teeth better than a more expensive amalgam. Dr. Flagg makes no mention of copper amalgam in his work on Plastics, although he has great faith in copper as a constituent of an alloy for amalgams. I am told that in his lectures before the students of the Philadelphia Dental College last winter, speaking of copper amalgam, he exclaimed, with emphasis, that "it was the stuff!" Dr. W. D. Miller, of Berlin, Germany, in his "Fermentation in the Human Mouth," as published originally in the INDEPENDENT PRACTITIONER, gives the result of a great number of experiments which tend to show that the "only filling at present in use which exerts a continual antiferment action upon the walls of a tooth and its immediate surroundings is the old copper amalgam; not only that, but the very substance of the tooth containing such a filling itself becomes antiseptic," and goes on to say that "if our only object is to check the destruction of tissue by caries, there is no material at present in use with which this object may be so surely accomplished as with a good copper amalgam."

Ever since amalgam came into use as a material for filling decayed teeth, experimentalists have been endeavoring to overcome its numerous defects. With these we are all only too well acquainted, and from the fact of their being so universally known it may be inferred that, so far, the grand desideratum has not been reached. The most serious faults with which amalgams are accused are those

---

* *Dental Cosmos*, Vol. XXII, page 197.
† *Dental Cosmos*, Vol. XXII, page 197.
‡ *Dental Miscellany*, Vol. V, page 365.
§ *Dental Cosmos*, Vol. XXII, page 197.

of shrinkage and discoloration, and these two defects seem to be, as it were, antagonistic to each other. Amalgams which discolor the most show the least shrinkage. The effect of shrinkage in an amalgam plug is to allow the fluids of the mouth to gain access to the cavity, and decay to reassert itself. We know that certain classes of teeth are eminently predisposed to decay under any filling; how much more likely would they be to do so, when the filling itself is predisposed to allow it? Now, when we consider that about one-half the teeth in the mouth are practically out of sight, it is not such a great matter if they are, to some extent, rendered unsightly by the presence of a black filling, if they are thereby physically qualified to resist the advance of the enemy. How often do we see amalgam fillings that have been inserted in shallow, hypersensitive buccal cavities in lower molars, in a few months getting loose or bulging out. We cannot always attribute it to imperfect manipulation, because, perhaps, we notice it more in our own work than in that of others, and possibly have had the same thing occur repeatedly in the same cavity, although the utmost care had been taken in every step of the operation. Nor does it always occur in teeth of poor quality. We can only accuse the material in the filling itself of being in fault, and notwithstanding the fact that gold, tin or gutta-percha would not answer as well for the case in hand, we are reluctantly obliged to use them. Then is the time in a man's experience when he longs for a material which will be thoroughly reliable, and which he can place in the cavity with all confidence that it will stay there—that it will neither shrink nor ball up. I fill such cavities with copper amalgam, and when, after two or three years, I have occasion to examine them, I cannot discover the slightest sign of shrinkage or bulging. In fact, a great many of these fillings, properly finished up, remain in such perfect condition that after two years the edge cannot be felt when the point of an excavator is passed over it, the joint being so close. I would like to ask what other amalgam will stand such a test as that?

As copper amalgam is unchanging in its character, and becomes so very hard, it may be placed in cavities having only slight retaining points, with a reasonable expectation of its remaining. All shallow cavities out of sight, such as we find upon the buccal, lingual, or approximal surfaces of molars near the margin of the gum, may be advantageously filled with it. Rapidly decaying wisdom teeth and molars of a soft, chalky nature, seem to do better with a copper filling than any other. It may also be used under water, but gives better results if the cavity is kept dry. Having antiseptic properties, a layer of decayed dentine left under it is rendered comparatively harmless. As the margins of a copper filling are tight and impervious to the fluids of the mouth, no discoloration of tooth substance takes place, so that posterior cavities of badly decayed bicuspids, and even cuspids, may be filled with it, without danger of blackening or disfiguring any portion of the tooth-structure exposed to view. For the deciduous molars I know of no better filling than copper amalgam. As a general rule it is not practicable, nor is it good policy, to give as much time to the preparation of cavities in these teeth as we do for those of adults. From the mere fact of children's teeth decaying at so early an age, we are led to infer that dental operations must be kept up for a number of years at least, and our little patients must not be frightened or tired out by long, tedious operations at the very outset of their acquaintance with dental treatment. A filling which can be quickly and painlessly inserted in a cavity that has not been too carefully prepared, at the same time preventing decay better than gold, and one which is far more durable than gutta-percha or the mineral cements, is a filling we should by no means despise.

In case some may wish to make a little copper amalgam, I will give my method of preparation. I may say there are several ways of preparing it, but the following I have found to be altogether the best: Nearly fill a tall, narrow glass beaker, or other vessel of suitable shape, with a weak solution of sulphate of copper, say one part of a saturated solution to two or three parts water. Pour in enough mercury to well cover the bottom of the glass, and stand a clean strip or plate of iron in the mercury, allowing the end to project above the glass. Sheet-iron, such as is used for stove-pipes, will do for a small quantity, but heavier pieces are better. Pure precipitated copper in a finely divided state will at once become deposited upon the iron, and soon we shall find the mercury uniting with the copper and gradually creeping up the iron until the whole surface is covered with a film of amalgam. If the iron is placed for a moment in a weak solution of sulphuric acid immediately before immersing in the copper bath, amalgamation takes place more rapidly.

It must be allowed to stand undisturbed until the change in the color of the solution shows that all the copper is precipitated. Then with a syphon draw off the liquid and renew the sulphate of copper. This proceeding may be repeated as long as the mercury takes up the copper. When all the mercury has become amalgamated, scrape off whatever amalgam adheres to the strip of iron, pour off the liquid, and turn the mass of amalgam into a mortar. Rub and wash it thoroughly, allowing a stream of water to fall upon it from a tap, cleaning out all the free metallic copper and scales of oxide of iron. As soon as it is as clean as it can be made, place it in a chamois skin and squeeze out the surplus mercury. Then the washing and grinding in the mortar must be repeated until the mass again becomes soft, when more mercury can be removed. The greatest care must be taken to remove all the little scales and grains of iron, or the amalgam will be dirty to work, and the best results from it cannot be obtained. When the amalgam has been well worked and all the mercury possible squeezed out, heat it gently in an iron vessel. The first time this must be done carefully, as steam from water which is retained in it becomes generated and the mass will explode, flying in all directions. When the amalgam begins to get soft, rub in a mortar and again squeeze out mercury. This heating, rubbing and squeezing must be repeated again and again, until very little mercury can be removed and the amalgam is found to set instantly, and get very hard. It may then be made into little sticks or pellets, and laid away for use. To use it, place the quantity required in an iron spoon and heat it over a flame until mercury begins to show like sweat upon the surface. Then crush and grind the mass in a small mortar, and work together in the hand. If too soft, squeeze in a piece of chamois skin, using a pair of pliers if necessary. One soon learns how soft or dry to make it in order to get the best results. Do not throw away any of the scraps remaining, as they may be used over and over again an indefinite number of times, seeming to improve by age. Be careful not to heat too much, as some of the mercury volatilizes, leaving pure copper, which becomes oxidized by the heat and makes the amalgam dirty.

A weak solution of sulphate of copper is used instead of the saturated solution, as the precipitate is much finer and the amalgam requires less rubbing to bring it to shape.

